# Retrograde labeling, transduction, and genetic targeting allow cellular analysis of corticospinal motor neurons: implications in health and disease

**DOI:** 10.3389/fnana.2014.00016

**Published:** 2014-03-26

**Authors:** Javier H. Jara, Barış Genç, Jodi L. Klessner, P. Hande Özdinler

**Affiliations:** ^1^Davee Department of Neurology and Clinical Neurological Sciences, Feinberg School of Medicine, Northwestern UniversityChicago, IL, USA; ^2^Robert H. Lurie Cancer Center, Feinberg School of Medicine, Northwestern UniversityChicago, IL, USA; ^3^Cognitive Neurology and Alzheimer's Disease Center, Feinberg School of Medicine, Northwestern UniversityChicago IL, USA

**Keywords:** corticospinal motor neuron, genetic labeling, retrograde labeling, motor neuron disease, upper motor neurons

## Abstract

Corticospinal motor neurons (CSMN) have a unique ability to receive, integrate, translate, and transmit the cerebral cortex's input toward spinal cord targets and therefore act as a “spokesperson” for the initiation and modulation of voluntary movements that require cortical input. CSMN degeneration has an immense impact on motor neuron circuitry and is one of the underlying causes of numerous neurodegenerative diseases, such as primary lateral sclerosis (PLS), hereditary spastic paraplegia (HSP), and amyotrophic lateral sclerosis (ALS). In addition, CSMN death results in long-term paralysis in spinal cord injury patients. Detailed cellular analyses are crucial to gain a better understanding of the pathologies underlying CSMN degeneration. However, visualizing and identifying these vulnerable neuron populations in the complex and heterogeneous environment of the cerebral cortex have proved challenging. Here, we will review recent developments and current applications of novel strategies that reveal the cellular and molecular basis of CSMN health and vulnerability. Such studies hold promise for building long-term effective treatment solutions in the near future.

## Introduction

Our expertise in the precise control of fine movement sets us apart from other mammals. Voluntary movement is initiated, modulated, and controlled via a very complicated neural network, called the motor neuron circuitry, which includes neurons and cells that are located both in the cerebral cortex and the spinal cord. The output of neuron function is manifested by muscle contraction leading to precise movement of the legs, arms, and hands. It is this circuitry that helps define us as human beings by giving us a unique advantage to build and create tools and to express ourselves.

Since cognitive abilities are reflected in our actions, it is unreasonable to think that only one neuron type in the brain would be responsible for movement. Among all other neuron types in the cerebral motor cortex, however, one neuron population stands out with its unique abilities and function. These neurons are characterized by: (1) a large pyramidal cell body, (2) a single apical dendrite that extends toward layer I displaying major branching and arborization, especially within layer II/III, (3) numerous basal dendrites arising from the basolateral surface, and most impressively (4) a very long axon that projects toward spinal cord targets (Molnar and Cheung, 2006; Ozdinler and Macklis, 2006; Molyneaux et al., 2007). These neurons, known as Betz cells in humans, are located in layer V of the motor cortex. They are also referred to as the upper motor neurons, corticospinal neurons, and corticospinal projection neurons. We prefer using the name corticospinal motor neurons (CSMN) due to their unique ability and function and to emphasize their role within the motor neuron circuitry.

CSMN are special neuron populations in our cerebral cortex that can collect, integrate, translate, and transmit both the excitatory and the inhibitory cortical inputs as one single message to long distance spinal cord targets. This distinct ability allows them to act as the “spokesperson” of the cerebral cortex for the motor function. Without CSMN, especially in humans, the connection between the cerebral cortex and the spinal cord would be greatly impaired. Therefore, to emphasize the importance of cortical input to the motor neuron circuitry, we think the word “motor” is necessary when naming these long distance projection neurons of the cerebral cortex.

**Figure 1:**
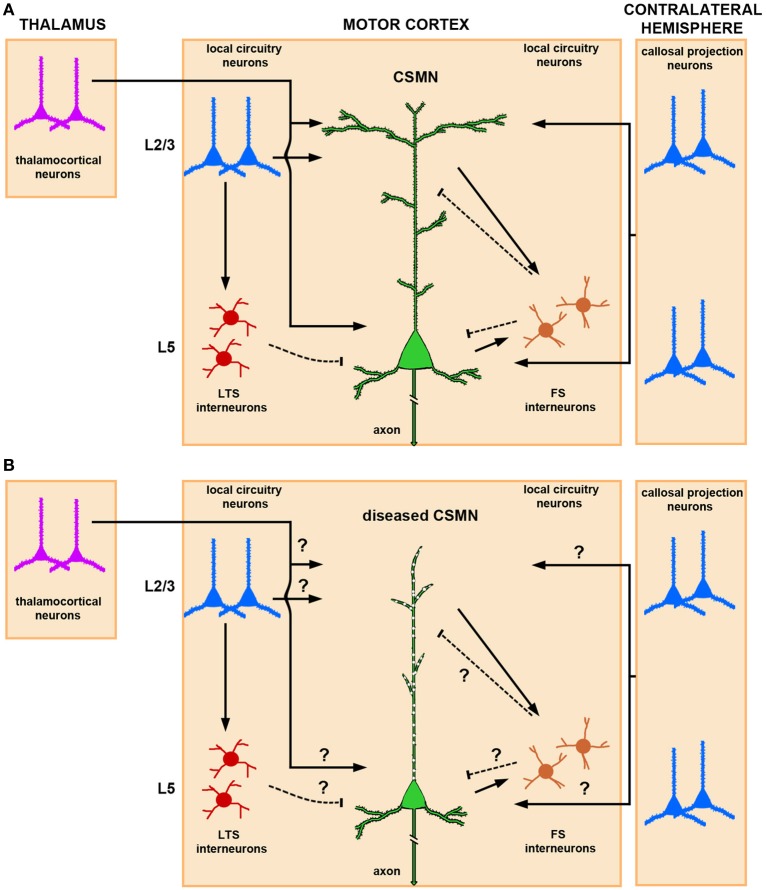
**Schematic and simplified representation of CSMN modulation by local neuronal circuitries and long-range projection neurons in normal physiological conditions and in disease**. **(A)** Thalamocortical projections provide excitatory input to CSMN. The site of neuronal modulation is suggested to be mainly within layer II/III and layer V. CSMN are also heavily modulated by both ipsi and contralateral callosal projection neurons that are mainly located within layer II/III and layer V of the cerebral cortex. Local circuitry neurons also modulate CSMN activity either directly or via an interneuron. In particular, LTS (low-threshold-spiking) and FS (fast-spiking) interneurons are differentially involved in the inhibitory microcircuit. While LTS interneurons receive interlaminar input from layer II/III projection neurons, FS receive intralaminar input from CSMN illustrating a possible distinct mechanism for disynaptic CSMN inhibition. Excitatory inputs impinge mainly upon the spines that are located on the dendrites located in layer II/III and layer V, and inhibitory input is conveyed mainly on the spines that are directly located on the apical dendrite and on a subset of spines that are present on basal dendrites. **(B)** In ALS, CSMN show vulnerability and undergo progressive degeneration. At pre-symptomatic stage (i.e., P60) CSMN display major spine loss within apical, but not basal, dendrites and apical dendrites undergo massive vacuolation and lose cytoarchitectural integrity. Such cellular defects would have profound impact on CSMN modulation. How circuitries are affected in disease require further investigation. Excitatory and inhibitory input is denoted by lines and dashed lines, respectively.

CSMN are heavily modulated by local neuron circuitry and long distance projection neurons, including, but not restricted to, thalamocortical neurons and callosal projection neurons (CPN) (Figure 1A). While details are still emerging about the timing and extent of CSMN regulation, anatomical studies suggest that the major excitatory input to CSMN is mediated by neurons located in layer II/III and layer V of the motor cortex (Thomson and Lamy, 2007; Shepherd, 2011). Thalamacortical neurons are important in carrying cognitive and sensory information to CSMN via neuronal networks that include cerebellum and basal ganglia (Clasca et al., 2012). The heterogeneity of these neurons in terms of somatodendritic morphology, axonal branching, and laminar specificity is of great importance. Subtle differences within the same neuron population could account for the variations of thalamacortical input to CSMN.

Another excitatory input is provided by CPN, which include ipsi- and contralateral projections (Anderson et al., 2010). CPN are highly heterogeneous, ranging from small to medium size, and are primarily located in all layers of the cortex, but most prominently in layer V and II/III. They carry integrative circuitry information (Arlotta et al., 2005; Molyneaux et al., 2007). This neuron population is closely related to CSMN; CPN are born from the same progenitor pool, migrate together in the developing cerebral cortex, and a subset of CPN reside together with CSMN in layer V. However, CPN project to the contralateral side of the cerebral hemisphere and display a different function in cognition, perception, and higher-order thinking.

In addition to long distance projection neurons, local circuitry neurons also have direct input to CSMN. Such neuronal interactions are rather interesting, as they can be mediated both directly and through a secondary neuron population. There is also CSMN-CSMN interaction, as nearby CSMN communicate with each other (Kiritani et al., 2012). Such interactions, which mostly occur within layer V of the motor cortex, could serve both as feedback and feed forward loops (Figure 1A).

Modulation of CSMN function involves a delicate and continuous balance between excitatory and inhibitory inputs. Interneurons account for about 20% of the cortical neuron population (Ikrar et al., 2011), and they are subdivided according to their anatomical location, cell morphology, specific-marker expression, dendritic arborization, and targeting of different sites and compartments of the cortical neurons including CSMN (Markram et al., 2004). Fast-spiking (FS) and low-threshold-spiking (LTS) are two types of interneurons that are implicated in CSMN inhibitory pathways (Tanaka et al., 2011). FS neurons have a high number of local connections to CSMN when compared to LTS. Recent studies designed to dissect out inhibitory inputs converging to CSMN have shown that these FS and LTS differentially inhibit CSMN (Apicella et al., 2012). While interlaminar input of pyramidal neurons in the layer II/III via LTS neurons provides disynaptic inhibition to CSMN (layer II/III→layer V), intralaminar input to FS from CSMN (layer V→layer V) delivers inhibition to CSMN in a feed forward manner (Figure 1A).

The progressive degeneration of CSMN is accepted as one of the major characteristics of neurodegenerative diseases affecting voluntary movement that require CSMN input to the motor neuron circuitry. For example, hereditary spastic paraplegia (HSP) is best characterized by the progressive degeneration of CSMN (Fink, 2001). The disease manifests itself with stiffness in the legs, paralysis and motor function defects. Primary lateral sclerosis (PLS) is also characterized by CSMN death and corticospinal tract (CST) degeneration. However, in amyotrophic lateral sclerosis (ALS) both the spinal and cortical motor neurons progressively degenerate (Udaka et al., 1986; Brown and Robberecht, 2001), adding complexity to the disease (Eisen and Weber, 2001; Ravits et al., 2007). Although spinal muscular atrophy (SMA) has been characterized by prominent spinal motor neurons (SMN) degeneration, recent evidence also suggests the involvement of CSMN (D'Errico et al., 2013). Lastly, exome sequencing analysis of patients with HSP revealed numerous shared common pathways and molecular networks that are also involved in ALS as well as Alzheimer's disease and Parkinson's disease (Novarino et al., 2014). This is a remarkable finding as it links numerous disorders at a molecular level. CSMN are clinically important and relevant neuron population that deserve immediate attention to improve future therapeutic applications both in injury and disease. If we can understand the molecular mechanisms responsible for their cellular vulnerability and degeneration, then we may begin to unravel the basis of numerous disease pathologies.

Even though mechanisms of CSMN modulation by cortical neurons is beginning to emerge, it is still not clear how that cortical connectivity and communication is hampered during disease. Since CSMN receive most of their input from the apical dendrite, cellular degeneration, disintegration of apical dendrite and spine loss would have significant consequences on the transfer of cortical signals to the spinal cord targets (Figure 1B). This indeed could be one of the reasons for a dysfunctional motor neuron circuitry and voluntary movement defects.

While CSMN are a crucial component of the motor neuron circuitry, they are not equally central in all species (Lemon, 2008). In rodents, the CST projects through the striatum, internal capsule, pons and pyramidal decussation, and subsequently descends in the ventral part of the dorsal column (Jones et al., 1982; Stanfield, 1992; Terashima, 1995). In humans, these projections descend laterally, and more than 80% of the axon fibers that originate from the CSMN in the motor cortex connect directly with SMN in the spinal cord (Lemon, 2008). This difference in projection paths affects neuronal circuits and connections defining the speed, specificity, and mode of action. Humans are very dexterous with their hands, but are mostly vulnerable to any injury that damages the CST. CSMN numbers are very limited in the cerebral cortex and their cellular degeneration results in severe consequences and is central to numerous neurodegenerative diseases and injury. For example, CSMN health and connectivity are hampered during spinal cord injury. Lesions of the CST result in deterioration in speed, force, and movement coordination (Lemon, 2008). One of the reasons for long-term paralysis in patients is the cellular degeneration of CSMN and the impaired connection between the cerebral cortex and the spinal cord.

We enter a new era of very exciting times in CSMN biology. Historically, there were serious limitations that hindered detailed studies of CSMN. Their importance as a neuron population was not well appreciated, and applications that allowed their cellular analysis were not available. Recently, numerous novel techniques and approaches to help identify and visualize CSMN within the complex structure of the cerebral cortex have been developed. AAV-mediated gene delivery and novel reporter lines now have the potential to change the future of CSMN investigations. In this review, we will introduce and describe these innovative approaches and comparatively discuss their limitations and advantages for future cellular analysis and therapeutic applications.

### Retrograde labeling approaches

With their axons projecting to distant targets, projection neurons are one of the most polarized cells in the body and highly depend on axonal transport to maintain cellular homeostasis. Retrograde transport depends on neurons' ability to carry proteins and molecules from the tip of the axon all the way back to the soma. Early studies revealed the basics of this cellular process in the central nervous system (Lavail and Lavail, 1972). Soon after, the ability of axons to uptake and retrogradely transport tracers and molecular dyes was uncovered making it possible to label, identify, and visualize neurons of interest based on their projection path and the pattern of their target innervation (Lavail et al., 1973). Retrograde transport studies demonstrated the importance of growth factors for the development and maturation of neurons. For example, when retrograde transport of nerve growth factor (NGF) to the cell body was revealed, the field opened up to a new idea that this phenomenon was actually critically important for the maintenance of neuronal health and function (Paravicini et al., 1975; Stoeckel and Thoenen, 1975), and it was possible for the axon to retrogradely transport large target-derived macromolecules important for their survival and differentiation (Hendry and Hill, 1980). The potential consequence of axonal transport defects became an area of interest (McLeod, 1975) and continues to be so with the identification of its association with a vast majority of neurodegenerative diseases (Morfini et al., 2009). In addition, identification of intrinsic differences between axonal transport of proteins in sensory and motor axons was remarkable as it pointed out the specificities of this phenomenon in different neuron populations (Bisby, 1977).

The anatomical knowledge of the timing and extent of axonal elongation, as well as the neuron's ability to perform retrograde transport, formed the basis of the initial studies that discern a distinct neuron population among many other neuron types. Horseradish peroxidase (HSP) was one of the first reagents used to study retrograde axonal transport and to visualize the cell bodies of projection neurons (Bunt et al., 1974; Kristensson and Olsson, 1974; Lavail and Lavail, 1974). Tetanus toxin and 3H-proline were introduced as agents that can be retrogradely transported in the axon (Kunzle, 1977; Price and Griffin, 1977) and were widely used in retrograde labeling studies, especially those showing transsynaptic transfer of tetanus toxin. These studies generated interest in its use to determine neuronal connections (Schwab et al., 1979).

The first fluorescent retrograde labeling was performed using red fluorescent Evans blue and blue fluorescent DAPI-primuline injections dramatically improving visualization of cells and the extent of their branching (van der Kooy and Kuypers, 1979). Since then, numerous reagents with different fluorescent properties have been used to retrogradely label neurons. For example, the Bisbenzimide and “nuclear yellow” produced green and golden-yellow labeling, respectively. True Blue, Fast Blue, and Fluoro-Gold (FG) produced blue retrograde labeling (Kuypers et al., 1980). Availability of different dyes with different colors allowed double and triple labeling experiments to study the details of axonal projection paths and to reveal the identity of neurons (De Olmos and Heimer, 1980). The disadvantages of these early dyes were their diffusion and lack of sustained stability within the cell, which limited their use in connectivity mapping studies.

The use of modern fluorescent tracers, such as latex-based microspheres (Lanciego and Wouterlood, 2011) and FG (Catapano et al., 2002), have been the most common approach for projection neuron labeling. FG and microspheres are taken up by axons and retrogradely transported to the cell body by fast axonal transport. These dyes are mostly engulfed in lysosomes that fill the somata with stable fluorescence. For example, injection of fluorescent microspheres into the contralateral hemisphere of the motor cortex or spinal cord provided labeling of two distinct projecting neuron populations: CPN and CSMN, respectively (Catapano et al., 2001; Arlotta et al., 2005; Ozdinler and Macklis, 2006). More recently, a novel immunopanning method to culture CSMN combined injection of cholera toxin β (CTB) conjugated to fluorescent microspheres to target the axonal tract of CSMN (Dugas et al., 2008), and CSMN containing CTB were then immunopanned with anti-CTB antibody to yield pure populations of CSMN.

A new era of systems biology began to emerge in the 1970s as the connections among neurons that are located far apart were illuminated. Retrograde labeling and tracing studies began to reveal the unaccounted extent of connectivity in the central nervous system (Cull, 1975; Yorke and Caviness, 1975; Somogyi et al., 1979). Very systematic and well-defined investigations initiated the early stages of cortical connection mapping studies (Broadwell, 1975; Bunt et al., 1975; Liedgren et al., 1976; Walberg et al., 1976; Wise and Jones, 1976), laying the foundation for our current understanding and also identifying defects that occur in the presence of mutations in key genes (Caviness and Yorke, 1976). In addition, dual labeling approaches began to demonstrate the relationship between two different neuron populations located in the same nucleus suggesting that they can have different functions and mode of actions (Steiger and Buttner-Ennever, 1978).

Retrograde labeling approaches also helped investigations of different neuron populations that are located in various regions of the nervous system and project to different targets, including the muscle. Injection of HSP into the developing limb of the chick embryo enabled the first cellular labeling of developing spinal motor neurons (Oppenheim and Heaton, 1975) and the detailed analysis showed the timing and extent of their development and projection (Landmesser, 1978). Similarly, trochlear motor neurons were first visualized by retrograde labeling using HSP (Sohal and Holt, 1978). The early postnatal development of motor neurons located in the facial nucleus of the brainstem were also studied with similar retrograde labeling approaches (Olsson and Kristensson, 1979).

Most relevant to this review, the origins of the pyramidal tract were first determined by HRP retrograde labeling (Biedenbach and Devito, 1980), and early studies employing double labeling approaches demonstrated that in the cat, corticospinal neurons were also present in the sensorimotor cortex, especially in area 3a (Rustioni and Hayes, 1981). Similar experiments in rat demonstrated the somatotopy of corticospinal projection neurons and revealed that CSMN were located in layer Vb of the motor cortex extending within the somatosensory cortex. Most impressively, the neurons projecting to the cervical levels of the spinal cord were located further away from the midline whereas the neurons that projected to distal parts of the spinal cord were found closer to the midline (Ullan and Artieda, 1981). Projection patterns of neuron populations located in different areas of the brain, such as the red nucleus, were also studied using double labeling. These approaches revealed the complexity of the descending spinal pathways (Huisman et al., 1981) and originated many other studies to understand the development and establishment of connections in the motor neuron circuitry that control voluntary movement.

Early and seminal studies, in which the CSMN of the monkey were intracellularly filled with HRP and their anterograde axonal projections were studied in the spinal cord, showed very clearly that CSMN axons mainly terminate in lamina IX and make direct contacts with spinal motor neurons in the spinal cord (Shinoda et al., 1981). Species-specific differences in axonal path formations, target innervations, and circuitry building also began to emerge with the help of this approach (Jones and Leavitt, 1974). The development of the pyramidal tract and neuronal connectivity was studied in many different species, including the hamster, which can regenerate its pyramidal tract axons upon injury if it occurs early in development (Reh and Kalil, 1981). Retrograde labeling studies also enabled the comparative analysis of species differences. Numerous studies using cats, mice, monkeys, and hamsters demonstrated how these species-specific differences affect neural connections and overall networks (Tolbert et al., 1978; Beckstead et al., 1981).

The CST arises from CSMN located in layer V of the motor cortex. CSMN are born at embryonic day (E) 13.5 in the mouse and start migrating toward layer V of the motor cortex without a major axonal projection (O'Leary and Koester, 1993). By E17, CSMN axons reach the pons and CST axons enter the spinal cord by postnatal day P0 and continue to elongate until P14. By P14, even the most caudal targets in the spinal cord are innervated. This information proves to be very useful when labeling CSMN at different stages of their development. The other important information is related to cellular identity. Even though CSMN can be considered a “pure” neuron population, they are divided into subgroups that innervate different targets within the spinal cord: the cervical, lumbar, and thoracic regions. There are several differences in the CST among species; the anatomical location of CST fibers is in the dorsal columns of the spinal cord in mice, whereas in primates and humans CST fibers are mostly located in the lateral columns and about 10% descends ipsilaterally (Courtine et al., 2007). The CSMN projection field has been extensively studied in rodents. For instance, anterograde studies utilizing HRP have demonstrated the projection field of CSMN in the dorsal funiculus of the spinal cord in rats (Casale et al., 1988), These studies were further confirmed using biotin dextran-amine. CST fibers were found in on all levels of the spinal cord in rats (Brosamle and Schwab, 1997) and mice (Liang et al., 2011). The timing of CSMN axonal growth through the brain to the spinal cord has also been demonstrated with anterograde techniques (Canty and Murphy, 2008). Hence, the anatomical knowledge of axonal projections and their timing is valuable for labeling distinct projection neuron populations and for distinguishing different types of neurons that reside together in the cerebral cortex but project to different areas in the central nervous system.

**Figure 2:**
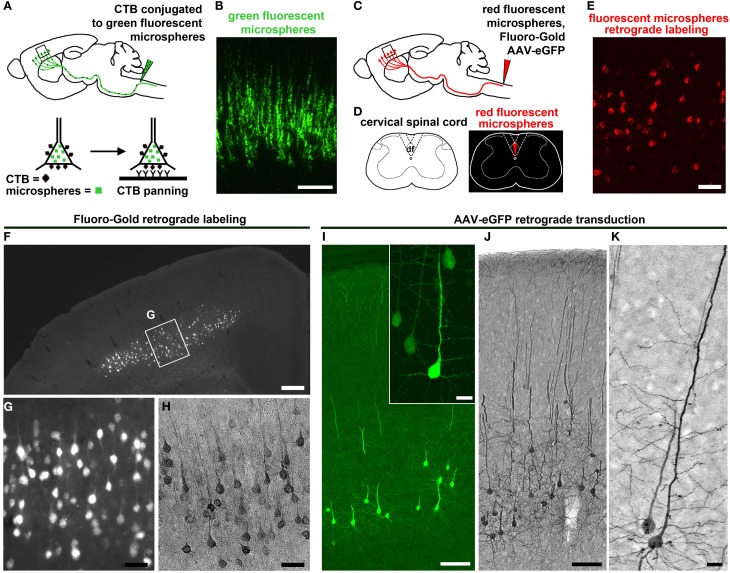
**CSMN retrograde labeling and transduction approaches. (A)** Schematic drawing of CTB injection conjugated to green fluorescent microspheres into the pyramidal decussation of the spinal cord. Retrograde labeled CSMN containing CTB on their surface can be isolated by immunopanning. **(B)** Enlarge inset of the motor cortex enlarged to the right. Modified from Dugas et al. (2008). **(C)** Schematic drawing of retrograde labeling of CSMN by injection into the CST of the cervical spinal cord (C2). **(D)** Injection is restricted to the CST that lies within the dorsal funiculus (df). **(E)** Enlarged inset of the motor cortex showing CSMN retrogadely labeled with red fluorescent microspheres. **(F)** FG retrograde labeling. CSMN are visualized throughout the motor cortex by direct analysis on fluorescent microscope. **(G)** Enlarged inset of CSMN in the motor cortex. **(H)** CSMN cell body and proximal apical dendrite structures are enhanced by FG immunocytochemistry using DAB. **(I)** AAV-mediated retrograde transduction. CSMN are visualized throughout the motor cortex by eGFP immunocytochemistry using fluorescence microscopy. Large inset of a representative CSMN in the right corner. **(J)** CSMN cytoarchitectural structure is enhanced by eGFP immunocytochemistry using DAB. **(K)** large inset of a CSMN shows details of CSMN cell body, basal dendrites, apical dendrite and spines. Scale bars: **(B)** = 100 μm, **(E)** = 50 μm, **(F)** = 500 μ m, **(G,H)** = 50 μ m, **(I)** = 100 μ m (inset = 20 μ m), **(J)** = 100 μ m, **(K)** = 20 μ m.

Understanding the details of CSMN connectivity also depends on retrograde labeling approaches to reveal the location of CSMN within the complex structure of the motor cortex. Retrograde labeling of CSMN in adult mice also facilitated studies during adulthood and when CSMN are affected in disease (Figures 2C–E). Studies using FG retrograde labeling demonstrated the presence of CSMN in layer V of the motor cortex under UV light (Figures 2F,G). In addition, FG visualization was enhanced by immunocytochemistry with DAB (Figure 2H). This allowed for visualization of CSMN somata and a portion of the proximal apical dendrite, depending on the concentration and elapsed time following FG injection. Using this approach, analysis of the hSOD1^G93A^ ALS mouse model has demonstrated that CSMN degeneration is pre-symtomatic and related to apoptotic mechanisms (Ozdinler et al., 2011). During ALS pathology, CSMN degenerate and there are patterns of cortical hyperexcitability that suggest dysfunction of CSMN in the cerebral cortex (Shepherd, 2013).

A wealth of information on CSMN biology has been generated using retrograde labeling approaches coupled with numerous applications. Since microspheres and FG fluorescently label cells, projection neurons can be purified using fluorescent activated cell sorting (FACS). In the case of CTB labeling, immunopanning is used providing a higher yield than FACS. Tissue culture approaches with CSMN purified using immunopanning approach revealed details of their survival requirements (Figures 2A,B) (Dugas et al., 2008), and FACS-purified CSMN displayed axon elongation in the presence of insulin-like growth factor 1 (IGF-1) and branching and arborization in the presence of brain-derived neurotrophic factor (BDNF) (Ozdinler and Macklis, 2006). Lastly, microarray analysis coupled with FACS-mediated purification of CSMN and CPN revealed the molecular signatures of CSMN and the genes that are important for their identification and early maturation (Arlotta et al., 2005).

Retrograde labeling coupled with electrophysiology are beginning to elucidate details of CSMN biology. For example, laser-scanning photostimulation in CSMN retrogradely labeled with fluorescent microspheres revealed unique intrinsic properties of CSMN when compared to CPN, such as differences in fast action potentials, firing rates, and hyperpolarization activated current modulation (Sheets et al., 2011; Suter et al., 2012).

Retrograde labeling techniques offer great advantages to study CSMN both *in vivo* and *in vitro*, but it falls short on providing the essential resolution to investigate the details of their cytoarchitecture. While the location of the cell body can be identified by the presence of fluorescent microspheres and the apex of the proximal apical dendrite can be visualized with FG labeling, they do not reveal cellular details. Virus-mediated gene delivery is one potential solution to this limitation.

### AAV-mediated retrograde transduction

Adeno-associated viruses (AAV) have been considered for their potential use in future therapeutic applications due to their low toxicity and ability to transduce a wide variety of cells (During and Leone, 1995; McCown, 2005; Han et al., 2010). They have been widely used to deliver genes of interest into the cerebral cortex of mice and rats for a variety of neurodegenerative diseases, such as Parkinson's disease (Mandel et al., 1997; Wang et al., 2002a) and ALS (Wang et al., 2002b; Boillee and Cleveland, 2004; Yamashita et al., 2013; Keifer et al., 2014). Because of their application, mechanistic insights into the pathologies that cause these diseases have been uncovered (Tatom et al., 2009; Langou et al., 2010; Decressac et al., 2012; Davies et al., 2013).

AAV have the potential to transduce a wide variety of cells and neurons. One way to achieve specificity is by retrograde transduction via injection into an axon tract selectively targeting only the cell bodies of neurons projecting through that tract. AAV-mediated retrograde transduction requires binding of the AAV to specific receptors on the axon surface and internalization through a receptor-mediated mechanism. Another way to achieve specificity is by viral vector capsid engineering. Recent advancements involving the discovery and generation of new AAV serotypes offer potential applications for gene delivery in distinct neuron populations (Weinberg et al., 2013). This is especially important considering that in neurodegenerative diseases distinct neuron populations show primary vulnerability and undergoes neurodegeneration. The ability to target the neurons that are vulnerable, without affecting other neurons, cells, and different circuitries, would be critically important for building long-term and effective therapeutic approaches. Here we will focus on the use and application of AAV on the genetic modulation of motor neurons and the components of the motor neuron circuitry.

One of the pioneering works came from studies demonstrating the ability to genetically label a distinct set of SMN by introducing AAV into the muscle fibers they innervate (Martinov et al., 2002). These studies were important as they show that AAV injected to the muscle can retrogradely transduce SMN, and most importantly distinct set of motor neurons are transduced based on their projection field. AAV that encode a gene of interest, such as glial-derived neurotrophic factor (GDNF), were then delivered to the muscle fibers to enhance growth factor expression in a distinct set of SMN (Lu et al., 2003), showing that it is indeed possible to use AAV to retrogradely transduce SMN and induce selective gene expression.

Neurotrophic growth factors that play key roles in the survival of motor neurons, including IGF-1, vascular endothelial growth factor (VEGF), GDNF, and granulocyte-colony stimulating factor (G-CSF), have been considered for ALS therapies (Hester et al., 2009). AAV2-IGF-1 injected into the muscle of the hSOD1^G93A^ mouse retrogradely transduced SMN, increased life span, and decreased disease progression were very encouraging (Kaspar et al., 2003). Introduction of IGF-1 into the deep cerebellar nuclei, a region of the cerebellum with extensive brainstem and spinal cord connection, via AAV suggested that the reduction of ALS pathology was due in part to its activity on non-neuronal cells in the CNS (Dodge et al., 2008) as it attenuated the release of tumor necrosis factor-alpha (TNF-α) and nitric oxide.

AAV-mediated gene delivery approaches have been increasingly used to modulate gene expression in SMN of ALS mouse models. Most recently, intramuscular delivery of the AAV6 encoding silencer shSOD1 RNA transduced SMN but failed to alter disease course in the hSOD1^G93A^ mice (Towne et al., 2011). However, these approaches have significant limitations as they only target the SMN that innervate specific muscle groups. Intrathecal injections releasing AAV6 or AAV9 serotypes at the level of the lumbar spinal cord transduced SMN throughout the spinal cord (Snyder et al., 2011), and intraspinal delivery of AAV-G-CSF showed a high transduction efficiency in SMN with modest improvement in the motor function as well as delayed disease progression and survival (Henriques et al., 2011).

Gene transduction in the cerebral cortex has also been considered via intracortical injections using different AAV serotypes. Different regions of the cerebral cortex have been transduced with a wide variety of AAV serotypes only to reconfirm the heterogeneity and the cellular complexity of the cerebral cortex and to realize that it is not be possible to transduce distinct neuron populations with one serotype (Burger et al., 2005; Hutson et al., 2011). Understanding the identity of neurons that are transduced in the cerebral cortex has not been easy, but investigating their axonal projection tracts might suggest their identity. For example, detection of transduced axons in the corpus callosum suggests that transduction of at least a subset of CPN as they cross the corpus callosum when projecting to the contralateral side of the cerebral cortex. Similarly, detection of transduced axons within the CST suggests transduction of subcerebral projection neurons in the cerebral cortex. Injection of AAV1-eGFP into the motor cortex resulted in visualization of GFP^+^ axon fibers within CST, suggesting that a subset of CSMN were transduced, and the identity of transduced neurons were further demonstrated with paired retrograde labeling (Hutson et al., 2011). Taking advantage of the retrograde transport capabilities and based on the hypothetical presence of specific receptors for different AAV serotypes, we have recently reported that AAV2 shows high levels of retrograde transduction efficiency in CSMN (Jara et al., 2012).

When compared to retrograde labeling techniques, AAV-mediated gene delivery is far more powerful in clarifying cytoarchitectural details. In addition, this novel technique has brought several improvements to previously reported methods by combining retrograde labeling and transduction applications to the study of CSMN. Retrograde labeling of CSMN, using dyes and microspheres, enables visualization of their cell bodies but fails to reveal the shape and length of dendritic spines. Even though subtle, these details could be particularly important for neuron function and connectivity. Since spines are the sites of active neuronal communication, their modulation, degeneration, and progressive loss could have implications on disease pathology. Indeed, there is now building evidence to suggest a link between spine morphology and a wide variety of neurological diseases (Penzes et al., 2011). Expressing eGFP gene selectively in CSMN using AAV-mediated retrograde transduction of the CST allowed exclusive, homogenous expression of eGFP within CSMN and analysis of neuronal structures (Figures 2I–K). AAV-mediated gene delivery demonstrated early and selective apical dendrite degeneration in the hSOD1^G93A^ ALS mouse model (Figure 1B). This finding was important because for the first time it deciphered the cellular events that occur in CSMN. Specifically, apical dendrited were filled with vacuoles and spines were lost or vastly degenerated. These defects occurred at P60, a time when SMN begin to show signs of cellular degeneration, suggesting that both upper and lower motor neurons degenerate together in synchrony. Neuronal degeneration was previously thought to develop in a sequence of events that started in the neuromuscular junction spreading to the SMN and CST until finally reaching the CSMN cell body. However, the use of AAV-mediated gene delivery provided a very detailed observation of CSMN and uncovered details about cellular degeneration within the motor neuron circuitry that occurs in ALS. Absence of spines and disintegrating apical dendrites early in the disease may suggest that CSMN do not receive proper input from other cortical neurons that modulate their activity and this could be one of the factors contributing to the observed defects in the motor neuron circuitry. The importance of early apical dendrite degeneration deserves much attention as this is the area CSMN activity is heavily modulated (Shepherd, 2013), and could be one of the underlying causes for circuitry defects and motor dysfunction (Figure 1B).

Using AAV-mediated gene delivery as a potential therapeutic application to facilitate the repair and survival of CSMN in neurodegenerative diseases and after spinal cord injury is a provocative idea. Retrograde transduction also provides the means to study different subsets of CSMN by injecting into different regions of the spinal cord. For instance, cervical and lumbar injections can be utilized to study neurons that project to different spinal cord targets. However, in order to be successful, this approach would necessitate improvements in transduction specificity. There are two main approaches to increase specificity: one is the use of engineered capsid proteins and different serotypes, and the other is the choice of the promoter used to derive gene expression. Therefore, a better understanding of the serotype(s) that transduce CSMN and the use of engineered capsid proteins or different promoters to drive gene expression would improve selective transduction.

Even though selective targeting using retrograde labeling in mice is important, novel approaches need to be developed for selective CSMN transduction in the motor cortex of patients. In mice, the CST lies within the dorsal funiculus of the spinal cord, but in humans the majority of the CST lies within the lateral columns, limiting direct access. This anatomical difference of axon projection path is particularly important. The modulation of genes only in SMN may not be sufficient to implement a long-term and effective treatment strategy in ALS and other related motor neuron diseases. Therefore, approaches to transduce CSMN, in addition to SMN, need to be developed.

AAV approaches are not without their limitations. One disadvantage is related to the variations introduced by the surgical techniques. AAV transduction has very low levels of toxicity, but studies have revealed that intracranial injections might elicit an immune response (Weinberg et al., 2013). AAV have small packaging capacity (<5 kb) that might limit genes of interest. In addition, the timing of intervention and concentration to achieve a therapeutic effect might be challenging. We anticipate that in the future AAV engineering will offer new serotypes with novel properties that might facilitate the transduction of CSMN upon direct injection into the motor cortex. Interestingly, several clinical trials for other neurodegenerative diseases have explored the possibility of intracranial injections (Hester et al., 2009) suggesting that in the future similar approaches could be adapted for transduction of Betz cells in patients. However, prior to any meaningful cellular repair study, the underlying molecular causes of cellular vulnerability and degeneration need to be understood.

### Genetic labeling of distinct neuron populations

Genetic labeling allows visualization of cells with spatiotemporal resolution *in vivo*. Compared to surgical approaches, genetic labeling has several advantages such as reducing variability from subject to subject, experiment to experiment and lab-to-lab, ensuring reproducibility of findings. Potential limitations of genetic labeling are the limited availability of unique genes/markers for specific cell types and the alterations in gene expression. Certain genes undergo spatiotemporal alterations throughout development and even environmental factors may affect control of gene expression by epigenetic mechanisms. However, advantages of genetic labeling outweigh any disadvantages and are widely used (Huang and Zeng, 2013).

There are three main different ways to achieve genetic targeting: (1) conventional/bacterial artificial chromosome (BAC) transgene (Schmidt et al., 2013), (2) gene knock-in (Taniguchi et al., 2011), and (3) gene/enhancer trapping (Leighton et al., 2001; Kelsch et al., 2012). Conventional or BAC transgene approaches use a transgene cassette, or BAC, to introduce the promoter/enhancer regions of the gene of interest to drive expression of a reporter which mimics endogenous expression patterns. They are randomly inserted in the genome, which may cause variations in expression patterns among founder lines. Gene knock-in relies on inserting the reporter gene in the endogenous location of the gene of interest to fully recapitulate endogenous expression patterns, however it is technically more challenging and expression of the target gene itself may be altered even using an internal ribosome entry site (IRES) sequence. In enhancer trapping, a reporter driven by a minimal promoter is randomly inserted in the genome, and local enhancers near the insertion site then determine expression of the reporter.

Discovery of green fluorescent protein (GFP) (Tsien, 1998) in 1962 was a significant milestone for cell and molecular biology, and it was granted the Nobel Prize in Chemistry in 2008 (Weiss, 2008). It was not long before GFP was used as a marker for gene expression in eukaryotes (Chalfie et al., 1994). Since then, there has been an explosion of its spectral variants with a wide variety of brightness and colors (Shaner et al., 2005). Transgenic mice expressing GFP under control of the cytomegalovirus (CMV) promoter allowed study of the central nervous system at a level of single individual neuron resolution (van den Pol and Ghosh, 1998). Spectral variants of GFP (yellow, YFP; cyan, CFP; and red, RFP; collectively called XFP) expressed in transgenic mice under the control of Thy-1 promoter allowed imaging and identification of numerous subsets of neurons (Feng et al., 2000). More recently, using a cre/lox recombination system to express multiple distinct colors in diverse genetically labeled neurons gave rise to the “Brainbow” mice (Livet et al., 2007; Cai et al., 2013).

Numerous reporter mouse lines have been generated using genetic targeting methods and extensively used to study various cell types in the mouse CNS, such as layer V pyramidal neurons (Feng et al., 2000; Yu et al., 2008), cortical interneurons (Chattopadhyaya et al., 2004; Lopez-Bendito et al., 2004; Taniguchi et al., 2011), basal ganglia (Gerfen et al., 2013), spinal cord motor neurons and interneurons (Wichterle et al., 2002; Miles et al., 2004; Chang and Martin, 2011). Of these, Thy-1 XFP mice are of particular interest as they allow analysis of labeled cells at a very fine detail, such as dendritic spine dynamics *in vivo* (Grutzendler et al., 2002; Oray et al., 2004; Dombeck et al., 2007). Moreover, they have been used to study mouse models of neurodegenerative diseases at a cellular level, such as dendritic spine loss in triple transgenic Alzheimer's disease mice (Bittner et al., 2010) and motor neuron pathology in the experimental autoimmune encephalomyelitis, and mouse model of multiple sclerosis (Bannerman et al., 2005). Thy1-YFP mice have extensively been used to study motor systems, not only SMN in mouse models of ALS (Schaefer et al., 2005; Wong et al., 2009), but also CSMN (Richter and Roskams, 2009) and their axons in spinal cord injury (Bareyre et al., 2005) and an ALS mouse model (Ozdinler et al., 2011).

Another reporter line extensively used to study motor systems is HB9-GFP, which labels about 90% of SMN in the developing embryo (Wichterle et al., 2002) but only about half of large Choline acetyl transferase (ChAT)^+^ SMN in the ventral horn of the adult (Chang and Martin, 2011). γ-SMN innervating muscle spindles are GFP^−^ (Shneider et al., 2009). HB9-GFP mice have been used to follow differentiation of mouse embryonic stem cells into motor neurons (Wichterle et al., 2002; Wu et al., 2012). HP9-GFP^+^ embryonic stem cell-derived SMN co-cultured with astrocytes expressing hSOD1^G93A^ displayed increased cell death and shorter axon length (Dodge et al., 2008). HB9-GFP BACs have also been used to characterize human embryonic stem cell-derived motor neuron lineages (Placantonakis et al., 2009) or facilitate FACS purification of human spinal motor neurons from embryonic stem cells (Singh Roy et al., 2005). Human embryonic stem cell-derived motor neurons identified by HB9-GFP expression showed that SMN with hSOD1 mutations displayed reduced survival and shorter axons (Karumbayaram et al., 2009). Primary and embryonic stem cell-derived motor neurons from HB9-GFP mice have been used to study neurodegenerative properties of human SOD1 mutations, showing glia and astrocytes with SOD1 mutations induce neurodegeneration of co-cultured SMN even when they do not carry the mutation themselves (Di Giorgio et al., 2007; Nagai et al., 2007). HB9-GFP mice have been used to study (1) motor axon guidance *in vivo* by Semaphorin signaling (Huber et al., 2005), (2) *in vitro* by GDNF chemoattraction and ephrinA signaling (Dudanova et al., 2010) during normal development, (3) targeting of regenerating motor axons *in vivo* by polysialylated NCAM in the adult (Franz et al., 2005), and (4) motor axon development *in vivo* in mouse models of disease such as SMA (McGovern et al., 2008).

**Figure 3:**
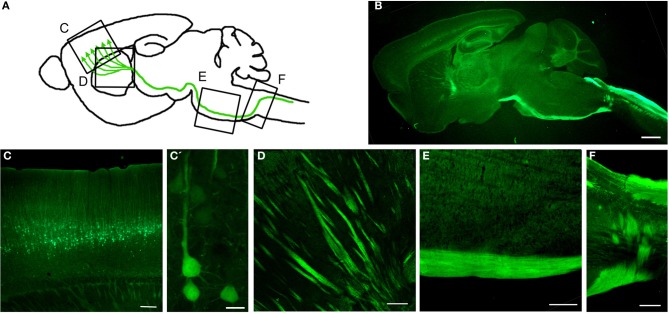
**Genetic Labeling of CSMN. (A)** Schematic drawing of genetic labeling of CSMN by eGFP expression under control of UCHL1 promoter. **(B)** A representative sagittal section of a P120 mouse brain showing eGFP^+^ CSMN and their axons. **(C)** In motor cortex, eGFP^+^ CSMN can be seen in layer 5a and 5b. **(C')** Higher magnification of individual representative eGFP^+^ CSMN in the motor cortex. **(D–F)** eGFP^+^ axons of CSMN projecting through the striatum **(D)**, pons **(E)**, and pyramidal decussation **(F)**. Scale bars: **(B)** = 1 mm, **(C,D** and **E)** = 200μ m, **(C')** = 20μ m, **(F)** = 500μ m.

Gene Expression Nervous System Atlas (GENSAT) database has been invaluable in providing a detailed library of hundreds of distinct, genetically defined cell populations from engineered mice utilizing BAC (Gong et al., 2003, 2007; Doyle et al., 2008; Schmidt et al., 2013). To date, there are almost 400 publications that use GENSAT mice or BACs (www.GENSAT.org). Using this approach, we generated the UCHL1-eGFP mice in which the UCHL1 gene promoter was used to drive eGFP expression (Figure 3) (Yasvoina et al., 2013). CSMN identity of eGFP^+^ neurons was confirmed by retrograde labeling, molecular marker expression profile, electrophysiology, cortical circuit mapping, and mouse genetics studies. CSMN in the motor cortex and their projections were genetically and stably labeled by GFP expression from P0 to P800. In the spinal cord, almost all ChAT^+^ SMN were eGFP^+^ at birth but by P30 eGFP expression became restricted to a mixture of small α- and γ-SMN that were resistant to degeneration in motor neuron diseases, such as ALS. Crossing this reporter mouse with hSOD1^G93A^ ALS mouse model (Gurney et al., 1994) generated hSOD1^G93A^-UeGFP mice, which allowed detailed study of CSMN health. We observed a progressive degeneration of eGFP^+^ CSMN, as previously reported (Ozdinler et al., 2011), with apical dendrite vacuolation and presence of autophagosomes, suggesting an ongoing intrinsic cellular degeneration. This novel reporter mouse model for ALS, now allows the detailed cellular analysis of CSMN without the requirement of retrograde labeling surgeries, which was not possible before.

The main challenge of genetic labeling approaches remains identification of genes/promoters that distinctly and specifically labels a neuronal subpopulation of interest. Once such reporters have been identified and characterized, there are numerous advanced applications. Recent advances in microscopy allow imaging cells in live animals (intravital imaging) (Pittet and Weissleder, 2011; Weigert et al., 2013). *In vivo* imaging has recently been applied to study spinal cord regeneration (Laskowski and Bradke, 2013). Optogenetics approaches take advantage of channelrhodopsin-2, which can be genetically targeted to neurons of interest and used to manipulate neural activity with millisecond precision using light (Packer et al., 2013). This allows imaging and long-range circuit mapping of multiple cell types (Atasoy et al., 2008). Optophysiology also grants success in such studies (Smedemark-Margulies and Trapani, 2013). Recently, optogenetic reactivation of hippocampal neurons activated during fear conditioning was shown to activate fear memory recall induced by light (Liu et al., 2012). Mosaic analysis with double markers (MADM) uses a site-specific recombinase to catalyze inter-chromosomal recombination that creates sparsely labeled neurons only after recombination (Luo, 2007). Type, density, and timing of labeling can be controlled by tissue-specific expression of the recombinase. Genetically encoded fluorescent protein-based biosensors can be used to study a broad assortment of signaling molecules and networks (Depry et al., 2013). For example, fluorescent proteins can be used to monitor protein-protein interactions in living cells using fluorescent resonance energy transfer (FRET) microscopy (Stepanenko et al., 2011; Day and Davidson, 2012). A recent nanobody-based technology utilizes fluorescent proteins commonly used to label cells and neurons as scaffolds for cell-specific gene manipulation allowing selective modulation of diverse GFP-labeled cells across transgenic lines such as optogenetic probing of neural circuits (Tang et al., 2013).

Numerous techniques and applications are being developed simultaneously to target neurons of interest. Advances in genetic labeling of select sets of neuron populations coupled with numerous applications that allow their cellular and molecular analysis at different experimental conditions and disease settings will reveal the underlying mechanisms for proper cellular function as well as the molecular networks that are responsible for their selective vulnerability. This information will be the foundation for future effective treatments strategies in diseases that primarily affect distinct neuron populations at initial stages.

## Conclusion

Techniques allow detailed analysis, but it is our critical thinking that shapes the field and the future. Many important discoveries are made when approaches and critical thinking develop simultaneously. The definition of “disease,” the description of pathologies, and our understanding of symptom development have vastly changed in the last decade. In addition, numerous new techniques and applications, which allow studies that were not previously possible, have become available. These two critical components of innovation have the potential to shape the future of neurodegenerative diseases once understood and applied properly. We now have reasons to believe that numerous discoveries will emerge in the near future.

Recently, the word “spectrum” was introduced when describing numerous neurodegenerative disorders to highlight the blurred boundaries among diseases and place them under an umbrella of systems degeneration with many common features. This is an important shift from disease-based thinking toward a more mechanism-oriented understanding that emphasizes the common underlying pathologies that give rise to different forms of neurodegeneration. This paradigm shift in our understanding has an impact on defining pathologies, disease mechanisms, and symptoms. The neurons that show vulnerability and undergo progressive degeneration have moved center stage. We now realize the importance of understanding the cellular, molecular, and genetic basis of pathology at a cellular level. This change in critical thinking proved to be correct by the failures of clinical trials. The expectation for improved lifespan in mice to translate into success in ALS patient survival is now considered mostly unrealistic. Today, clinical companies are more cautious when making a decision to move forward, and seek more cell-based evidence that supports motor neuron survival (Genç and Ozdinler, 2013).

In recent years, there have been significant changes in the meaning of the word “symptom.” Previously, symptoms were described as signs of pathology that can be detected by visual examination or various tests. However, it became obvious that our ability to detect signs of pathology at a molecular, cellular, and systems level was different. Even when no obvious signs were present, the pathology was taking its course at a cellular level. If the cellular basis of disease causing pathologies was understood, then these findings would set the stage, not only for early detection of the disease, but also for the development and implementation of effective long-term treatment strategies. Therefore, it is important to detect symptoms at a cellular level before pathologies become evident. We need to develop new approaches to focus our attention on the neuron, on the cell that is affected and has become vulnerable. Because if we understand the molecular and genetic basis of neuronal vulnerability at a cellular level, then we will have the chance to understand the basis of neuronal pathology and systems degeneration.

Studying CSMN biology is especially challenging, not only because they are limited in numbers and not easy to identify, but also because the techniques, technologies, approaches, and model systems to study them are not easy to develop. Due to species differences, modeling CSMN degeneration in mice has intrinsic limitations. Even though a minor defect would lead to a motor dysfunction in patients, mice would not display an obvious defect. This could indeed be one of the reasons why the mouse models of motor neuron diseases do not show a prominent phenotype. Working in models without an obvious functional readout can be challenging. However, if the models are built at a cellular level, or if the focus is shifted from mice to the motor neurons in mice, then the picture and the scope of perception changes dramatically. At a cellular level, the CSMN in humans and in mice are almost identical. It is thus important to develop technologies that reveal the underlying factors that contribute to the cellular vulnerability and degeneration. As long as our focus is the neuron and not the mice, the information gathered from healthy and diseased neurons would be translational.

Understanding the controls over CSMN health is becoming very central to numerous neurodegenerative diseases. Here we reviewed the exciting progress in the study of upper motor neurons. Retrograde labeling is a prominent approach to identify the location of the neuron and to visualize its cell body. AAV-mediated retrograde transduction, on the other hand, is not only a powerful tool to deliver genes of interest, but also important to reveal details of cellular cytoarchitecture. However, both of these applications require surgery and the success of each experiment mostly depends on surgical expertise and mouse survival. Genetic labeling that intrinsically targets a distinct neuron population offers a solution for the potential limitations of retrograde labeling surgeries. Generation and characterization of the UCHL1-eGFP mouse, in which the CSMN are genetically labeled, has been pivotal for overcoming numerous important limitations by allowing *in vivo* visualization and cellular analysis of neurons that are vulnerable in neurodegenerative diseases.

With the advancements made in the study of CSMN biology, the future awaits numerous important discoveries. FACS purification coupled with *in vitro* culturing will allow novel drug screening and verification platforms using motor neuron health as a readout for success. This application has the potential to discover new molecules and compounds for future clinical trials. In addition, development of AAV-mediated gene delivery, together with the identification of new genes and pathways that are important for disease pathology will enable direct cellular therapies into the motor cortex of patients improving the survival of upper motor neurons and enhancing their connectivity. Identification of the cellular and molecular basis of CSMN vulnerability will be revealed, paving the way for understanding the cellular pathways, networks, and dynamics that are important to improve motor neuron health.

We live in very exciting times due to the numerous improvements in our critical thinking of neurodegenerative diseases, and the technologies that support discoveries are developing faster than ever. The new knowledge gathered from neurons of interest will enable development of novel effective long-term treatment strategies and has the potential to expedite new discoveries that will enable improved health in patients.

### Conflict of interest statement

The authors declare that the research was conducted in the absence of any commercial or financial relationships that could be construed as a potential conflict of interest.
